# Targeting Ovarian Cancer Cells Overexpressing CD44 with Immunoliposomes Encapsulating Glycosylated Paclitaxel

**DOI:** 10.3390/ijms20051042

**Published:** 2019-02-27

**Authors:** Apriliana Cahya Khayrani, Hafizah Mahmud, Aung Ko Ko Oo, Maram H. Zahra, Miharu Oze, Juan Du, Md Jahangir Alam, Said M. Afify, Hagar A. Abu Quora, Tsukasa Shigehiro, Anna Sanchez Calle, Nobuhiro Okada, Akimasa Seno, Koki Fujita, Hiroki Hamada, Yuhki Seno, Tadakatsu Mandai, Masaharu Seno

**Affiliations:** 1 Department of Medical Bioengineering, Graduate School of Natural Science and Technology, Okayama University, Okayama 700-8530, Japan; phrw1rm8@s.okayama-u.ac.jp (A.C.K.); hfzhmhmd@gmail.com (H.M.); kokooo.aung@gmail.com (A.K.K.O.); px934zug@s.okayama-u.ac.jp (M.O.); pirh7vbs@s.okayama-u.ac.jp (J.D.); jahangir-geb@sust.edu (M.J.A.); saidafify@s.okayama-u.ac.jp (S.M.A.); Tsukasa.Shigehiro@roswellpark.org (T.S.); 2 Laboratory of Nano-Biotechnology, Graduate School of Interdisciplinary Science and Engineering in Health Systems, Okayama University, Okayama 700-8530, Japan; maram@okayama-u.ac.jp (M.H.Z); pfgg90pn@s.okayama-u.ac.jp (H.A.A.Q.); okadan@okayama-u.ac.jp (N.O.); aseno@okayama-u.ac.jp (A.S.); 3 Center For Immunotherapy, Roswell Park Cancer Institute, Buffalo, NY 14263, USA; 4 Division of Molecular and Cellular Medicine, National Cancer Center Research Institute, Tokyo 104-0045, Japan; annasc.nit@gmail.com; 5 Okayama University Research Laboratory for Stem Cell Engineering in Detroit, Integrative Biosciences Center, Wayne State University, Detroit, MI 48202, USA; 6 Ensuiko Sugar Refining Co., Ltd., Tokyo 103-0012, Japan; koki_fujita@pearlace.co.jp; 7 Faculty of Science, Okayama University of Science, Okayama 700-0005, Japan; hamada@dls.ous.ac.jp; 8 Graduate School of Pharmaceutical Science, Tokushima University, Tokushima 770-8505, Japan; s20b028@sci.kusa.ac.jp; 9 Faculty of Life Science, Kurashiki University of Science and the Arts, Kurashiki 712-8505, Japan; ted@chem.kusa.ac.jp

**Keywords:** CD44, liposome, specific targeting, ovarian cancer, glycosylated paclitaxel, modified paclitaxel

## Abstract

Paclitaxel (PTX) is one of the front-line drugs approved for the treatment of ovarian cancer. However, the application of PTX is limited due to the significant hydrophobicity and poor pharmacokinetics. We previously reported target-directed liposomes carrying tumor-selective conjugated antibody and encapsulated glycosylated PTX (gPTX-L) which successfully overcome the PTX limitation. The tubulin stabilizing activity of gPTX was equivalent to that of PTX while the cytotoxic activity of gPTX was reduced. In human ovarian cancer cell lines, SK-OV-3 and OVK18, the concentration at which cell growth was inhibited by 50% (IC_50_) for gPTX range from 15–20 nM, which was sensitive enough to address gPTX-L with tumor-selective antibody coupling for ovarian cancer therapy. The cell membrane receptor CD44 is associated with cancer progression and has been recognized as a cancer stem cell marker including ovarian cancer, becoming a suitable candidate to be targeted by gPTX-L therapy. In this study, gPTX-loading liposomes conjugated with anti-CD44 antibody (gPTX-IL) were assessed for the efficacy of targeting CD44-positive ovarian cancer cells. We successfully encapsulated gPTX into liposomes with the loading efficiency (LE) more than 80% in both of gPTX-L and gPTX-IL with a diameter of approximately 100 nm with efficacy of enhanced cytotoxicity in vitro and of convenient treatment in vivo. As the result, gPTX-IL efficiently suppressed tumor growth in vivo. Therefore gPTX-IL could be a promising formulation for effective ovarian cancer therapies.

## 1. Introduction

Ovarian cancer ranks the eighth cause of cancer death in women worldwide annually estimated to be 151,900 out of 238,700 new cases [1]. In the US as one of the typical developed countries, it ranks the fifth cause of lethal tumors among women, accounting for the seriousness in female gynecological cancers [2]. The standard treatment of progressive ovarian cancer is surgical resections followed by systemic chemotherapy [3,4]. The National Comprehensive Cancer Network (NCCN) guideline shows the first line of chemotherapy for ovarian cancer encompasses the administration of carboplatin, paclitaxel (PTX), or the administration of these two.

PTX acts as an anti-cancer agent preventing cell division by promoting and stabilizing the assembly of microtubule structures [4,5,6]. Because PTX is highly hydrophobic, the mixture of Cremophor EL^®^ and ethanol has been adopted as a solvent for the commercial formulation known as Taxol. However, Cremophor EL^®^ may induce anaphylactoid and/or anaphylactic reactions in vivo [7]. Taxol treatment itself is followed by side effects such as hypersensitivity reactions, nephrotoxicity and neurotoxicity [8,9]. Therefore, these disadvantages of Taxol treatment should urgently be ameliorated by the development of a proper drug delivery system for PTX.

During the past half-century, liposome has been considered one of the most promising drug-carrier systems of PTX due to its versatility, intrinsic biocompatibility and potential variability [10]. The highly hydrophobic nature of PTX hinder the loading efficiency (LE) into the liposome to establish efficient liposomal formulation. Recently, we successfully developed liposomes encapsulating glycosylated paclitaxel (gPTX) in the hydrophilic-core [11]. gPTX is a PTX derivative with a glucose moiety coupled at the 7-OH radical [12], and this modification enhanced the hydrophilicity of PTX allowing practically different solubility in the solvents mixed solvent of Cremophor EL^®^, ethanol, and phosphate buffered saline (PBS); in 12:12:76 ratio (CEP) and EG (40% ethylene glycol). Exploiting the difference of the solubility we could prepare stable gPTX liposome (gPTX-L) with a sufficient amount of encapsulated drug. The outer layer of liposomes can be modified with coupled ligands targeting molecules localized on the cells membrane surface.

Active-targeting drug delivery with ligands as nanosystem have been utilized to optimize therapeutic efficacy and minimize systemic toxicity. Ligands for cell surface receptors highly expressed in tumor cell populations have provided a great specificity [13,14]. The cell membrane receptor CD44 could be one of the most promising candidates to be targeted [15,16,17,18]. CD44 is a receptor for hyaluronic acid type-1 transmembrane glycoprotein that is implicated in cell–cell and cell–matrix interactions and is associated with malignancy, particularly with metastasis promotion [19,20]. CD44 has also been considered as a cancer stem cell (CSC) marker in several malignancies of hematopoietic and epithelial origin [20], and is closely related with tumor progression and drug resistance [21,22,23] in several tumors including ovarian cancer [15,16]. Collectively, CD44 could be a suitable candidate of target molecule in ovarian cancer to apply drug delivery and minimize side effects.

In this study, we improved the preparation of gPTX-L to achieve higher encapsulation efficiency (EE) and designed gPTX-IL, gPTX-L bound to anti-human CD44 monoclonal antibody (anti-hCD44 MAb) to target CD44 overexpressing ovarian cancer cells evaluating the physical parameters of liposome and the efficacy of gPTX-IL targeting CD44 positive ovarian cancer cells.

## 2. Results

### 2.1. Expression of CD44 in Human Ovarian Cancer Derived Cells

**Figure 1:**
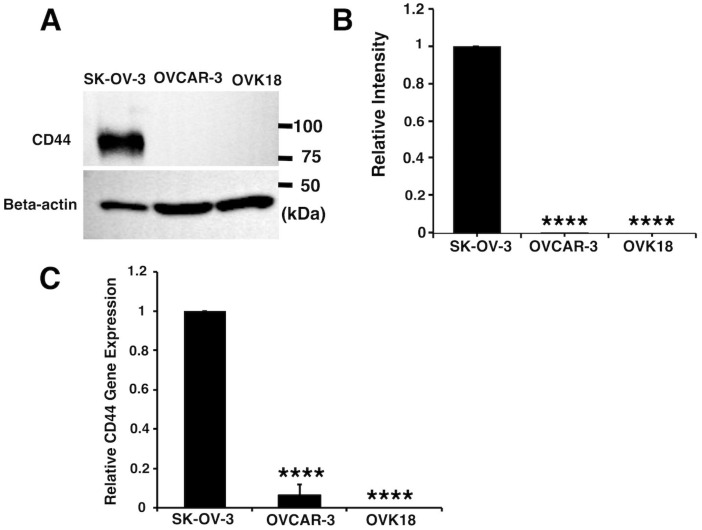
SK-OV-3 cells are overexpressing CD44. (**A**) Western blot analysis of human ovary cancer derived cells probed with anti-hCD44 MAb and human beta-actin antibody. (**B**) Relative intensity of the bands of CD44 to beta-actin in Western blot was densitometrically analyzed by ImageJ. (**C**) Relative gene expression levels of CD44 to glyceraldehyde-3-phosphate dehydrogenase (GAPDH) were analyzed by RT-qPCR. The data presented as the mean ± SD from three independent experiments. The statistical significance in mean values of more than two groups was determined using one-way analysis of variance (ANOVA) and Dunnet multiple comparison test using CD44 expression of SK-OV-3 cells as control; (****) *p* < 0.001.

First, we assessed the protein and mRNA expression levels of CD44 in the ovarian cancer cell lines SK-OV-3, OVCAR-3, and OVK18. Protein expression of CD44 detected by anti-hCD44 MAb, was found to be high in SK-OV-3 cells whereas was barely undetectable in OVCAR-3 and OVK18 (Figure 1A,B). The anti-hCD44 MAb showed the immunoreactive protein approximately at 85 kDa [24] (Figure 1A), which is attributed to the predominant isoform of CD44 known as the standard form. The mRNA expression of CD44 by reverse transcription-polymerase chain reaction (qRT-PCR) shown in Figure 1C confirm the result of CD44 protein expression in the same manner. Since the overexpression of CD44 was only found in SK-OV-3 cells line, we considered it as representative of CD44-positive cells. Likewise, the anti-hCD44 MAb was considered as a suitable ligand to target SK-OV-3 cells.

**Figure 2:**
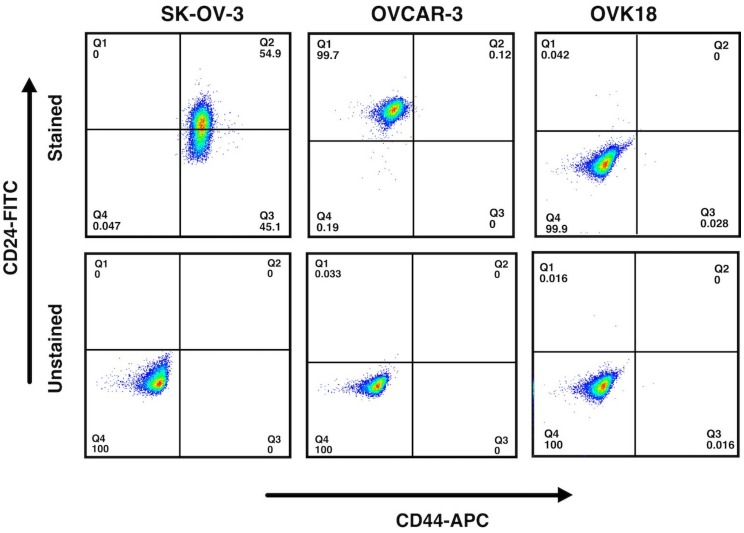
SK-OV-3 cells contain CD44^+^/CD24^−^ population as well as CD44^+^/CD24^+^ population. SK-OV-3, OVCAR-3, and OVK18 cells were analyzed by flow cytometry by staining for CD44 and CD24. The margins of CD24 and CD44 for each cell line were set up by non-stained cells as the negative control shown at the bottom of each analysis. Most of the population in SK-OV-3 cells were found CD44 positive.

Next, we confirmed the presence of CD44^+^ within the SK-OV-3 cells. The SK-OV-3 cells were characterized by CD44 and CD24 through flow cytometric analysis being compared with OVCAR-3 and OVK18 cells. The expression two antigens CD44 and CD24 has recently been used to explain the CSC population in breast cancer and ovarian cancer. The most population of SK-OV-3 cells exhibited CD44^+^, consisting of both CD44^+^/CD24^−^ and CD44^+^/CD24^+^ population while OVK18 cells showed only CD44^−^/CD24^−^ population and OVCAR-3 cells showed most CD44^−^/CD24^+^ population (Figure 2).

### 2.2. Sensitivity of Human Ovarian Cancer-Derived Cells to Glycosylated Paclitaxel (gPTX)

**Figure 3:**
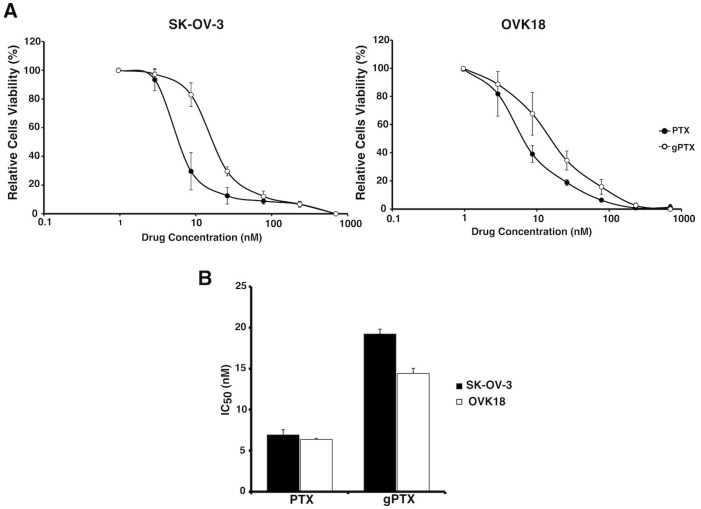
SK-OV-3 cells and OVK18 cells sensitive to paclitaxel and glycosylated paclitaxel. (**A**) Paclitaxel (PTX) and glycosylated paclitaxel (gPTX) sensitivity graph, cytotoxicity of both drug was assessed on SK-OV-3 and OVK18 cells by MTT assay after 72h drug treatment. (**B**) IC_50_ value of gPTX and PTX detemined by graph (**A**). The data presented as the mean ± SD from three independent experiment.

We assessed the anticancer effect of gPTX toward SK-OV-3 cells as CD44 positive cells and OVK18 cells as CD44 negative cells. In our previous report, gPTX was 3-fold weaker than PTX in breast cancer derived cells [11]. This observation was also consistent in ovarian cancer cells (Figure 3A,B). The reduced cytotoxicity should be caused by the increased of hydrophilicity of gPTX hindering penetration efficiency into the lipid bilayer of the cell membrane. However, the IC_50_ value of gPTX toward both cell lines is in the range of 15–20 nM, which means the cells are sensitive enough to give feasibility of using gPTX for ovarian cancer treatment. Moreover, encapsulation of gPTX into liposomes, which should confer gPTX with penetrability into the cytoplasm, and the specific ligand grafted on the liposome surface could help enhance the targeting potential minimizing systemic toxicity.

### 2.3. Potential Uptake of Liposome Conjugated with Anti-hCD44 MAb

**Figure 4:**
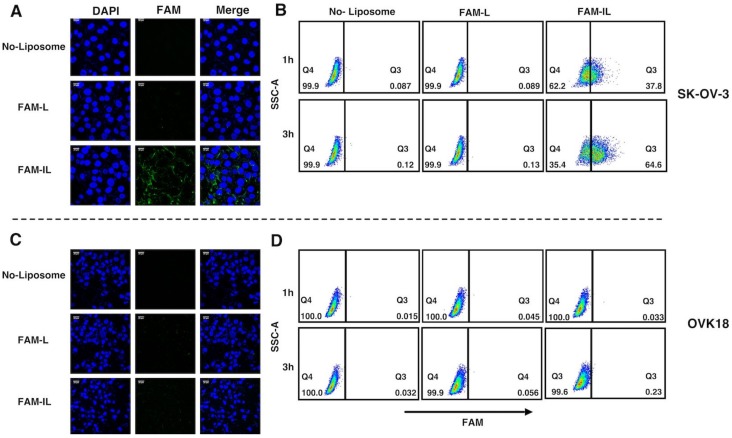
Immunoliposome enhanced cellular uptake in CD44 postive cells. (**A**,**C**) Confocal Microscopy image after 2 h treatment FAM-L and FAM-IL, Each scale bar shows 20 µm. (**B**,**D**) Flow cytometry analysis after 1 h and 3 h treatment FAM-L and FAM-IL. FAM-L and FAM-IL were evaluated for the cellular uptake in SK-OV-3 (**A**,**B**) and OVK18 (**C**,**D**), SSC-A is side scatter area. Data are representative of three replicates.

To assess the potential uptake of the liposomes conjugated to the anti-hCD44 MAb, we first prepared encapsulated 6-carboxyflourescent (FAM) into liposomes (FAM-L), which was conjugated with anti-hCD44 MAb (FAM-IL). The targeting potential of FAM-IL toward CD44 overexpressing cells, SK-OV-3 cells, was further assessed by confocal microscopic observation and flow cytometric analysis. The green fluorescence intensity of FAM between FAM-L and FAM-IL was equivalent and the green fluorescence observed in the cytoplasmic area was correlated with the intracellular uptake levels of liposome. After 2 h incubation at 37 °C of FAM-L and FAM-IL in the culture of SK-OV-3 cells, the uptake of FAM was evaluated under confocal microscopy (Figure 4A). Strong green fluorescent intensity of FAM was observed in SK-OV-3 cells when exposed to FAM-IL. According to the validation by flow cytometric analysis, SK-OV-3 cells incorporated FAM-IL in 1 h and kept up to 3 h (Figure 4B). In contrast, FAM-L did not show FAM fluorescence in OVK18 cells (Figure 4C,D), which showed no expression of CD44. These results imply that immunoliposomes targeting CD44 could effectively enhance the cellular uptake as compared with non-targeted liposomes.

### 2.4. Preparation and Characterization of gPTX-L and gPTX-IL

Preparation of liposomes encapsulating gPTX (gPTX-L) and those conjugated with anti-hCD44 MAb (gPTX-IL) followed the method previously described [11] except for the down-sizing procedure of liposomes. gPTX-L was prepared using remote loading encapsulation. Briefly, in the first step liposomes containing CEP were prepared by rehydrating lipid film with CEP, which consisted of cremophor, ethanol and PBS at the ratio of 12:12:76. Due to the high ratio of PBS, the hydrophobicity of CEP is not significant and we reported the similar ratio was sufficiently effective to encapsulate gPTX into liposomes, likewise CEP in our formula of liposome did not impair the stability of liposomes for 4 weeks after encapsulation of gPTX [11]. Finally, the liposomes encapsulated with gPTX were prepared by a remote loading method to facilitate efficient encapsulation of gPTX into the inner aqueous core of liposomes. This method is exploiting the difference of solubility of gPTX in the two solvents, 40% ethylene glycol (EG) and CEP. The gradient between the two solvents makes gPTX penetrate into liposomes efficiently because CEP increased the solubility of gPTX when compared to 40% EG. This remote loading process has previously been described as a driving force for active encapsulation of gPTX [11]. The down-sizing liposome process employed freeze-thawing process followed by extrusion through 100 nm polycarbonate membrane filter in place of sonication with a probe. We also modified gPTX-IL preparation by adding L-cysteine to block excess uncoupled maleimide [25,26]. This blockade was effective at stabilizing gPTX-IL preventing drugs from leakage. We could successfully prepare both gPTX-IL and gPTX-L as nanoparticles with homogeneous diameters of approximately 100 nm with polydispersity indexes less than 0.3, with negative zeta potential, and an improved encapsulation efficiency of gPTX inside with/without anti-hCD44 MAb (70–90%). The characters for the liposomes encapsulating gPTX in this study are summarized in Table 1.

**Figure 5:**
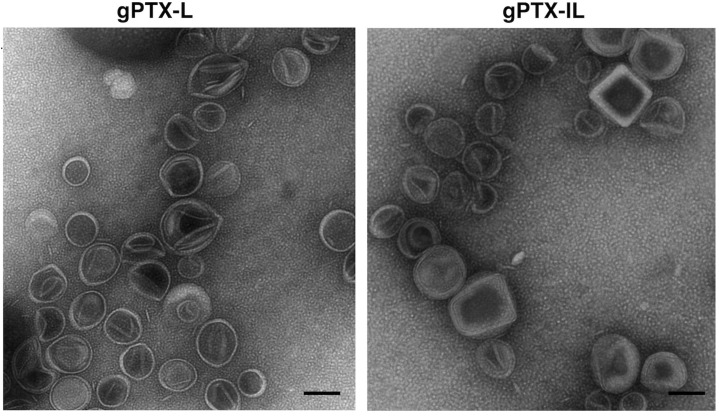
Transmission electron microscopy (TEM) images of liposome encapsulating gPTX. gPTX-IL showed unilamellar vesicles with diameter of approximately 100 nm similarly to gPTX-L. Each scale bar shows 100 nm.

Both gPTX-L and gPTX-IL were observed under transmission electron microscopy (TEM) (Figure 5). The images consistently indicated the diameter of gPTX-IL and gPTX-L at around 100 nm as measured by dynamic light scattering and the shape of each particle was spherical with a fairly smooth surface.

### 2.5. Cytotoxicity of gPTX, gPTX-L, gPTX-IL In Vitro

**Figure 6:**
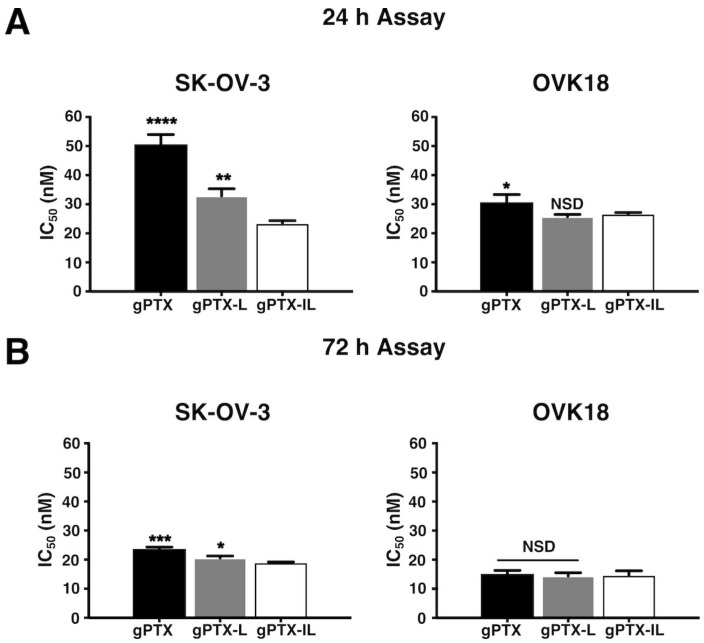
gPTX-IL exhibited the lowest inhibition concentration (IC_50_) in SK-OV-3 cells. In vitro cytotoxicity IC_50_ of gPTX in different formulation after 24 h (**A**) and 72 h (**B**) of exposure to SK-OV-3 cells and OVK18 cells was evaluated. The data presented as the mean ± SD (*n* = 3). The statistical significance in mean values of more than two groups was determined using one-way analysis of variance (ANOVA) and Dunnet multiple comparison test using IC_50_ value of gPTX-IL treatment as control, (*) *p* < 0.05; (**) *p* < 0.01; (***) *p* < 0.005; (****) *p* < 0.001; (NSD) no significant difference.

First, we assessed IC_50_s of gPTX as naked gPTX, gPTX-L, and gPTX-IL on SK-OV-3 and OVK18 cells comparing the two different exposure time 24 h (Figure 6A) and 72 h (Figure 6B). We found the exposure for 72 h of the three different formulations in vitro was too long to evaluate the IC_50_s since liposomes would fuse with cellular membrane independent of the antibodies of immunoliposomes when exposure time was long enough. In this context, gPTX-IL feasibly showed the IC_50_ of 19 nM in SK-OV-3 cells while naked gPTX and gPTX-L showed IC_50_s ranging from 20 to 22 nM, which were found in small differences to one another. To demonstrate the CD44 dependency of the immunoliposomes reducing the effect of the membrane fusion of liposomes, we thought that the drug exposure time should be shorter than 72 h. Antibody-oriented targeting of gPTX-IL successfully observed to be accumulated on the surface of SK-OV-3 cells due to the affinity of the antibodies in 24 h exhibiting the lowest IC_50_ of 23.4 nM while the IC_50_s of gPTX-L and naked gPTX were ranging between 30 and 50 nM. These results suggest that gPTX-IL should target CD44-expressing cells more efficiently than gPTX-L. Meanwhile, in both cells, naked gPTX and gPTX-L were still cytotoxicic even though they do not have targeting ability. As for the cytotoxicity of naked gPTX, the molecule is possibly internalized into the cell via passive diffusion due to its poor hydrophilicity.

### 2.6. Suppression of Tumor Growth In Vivo

**Figure 7:**
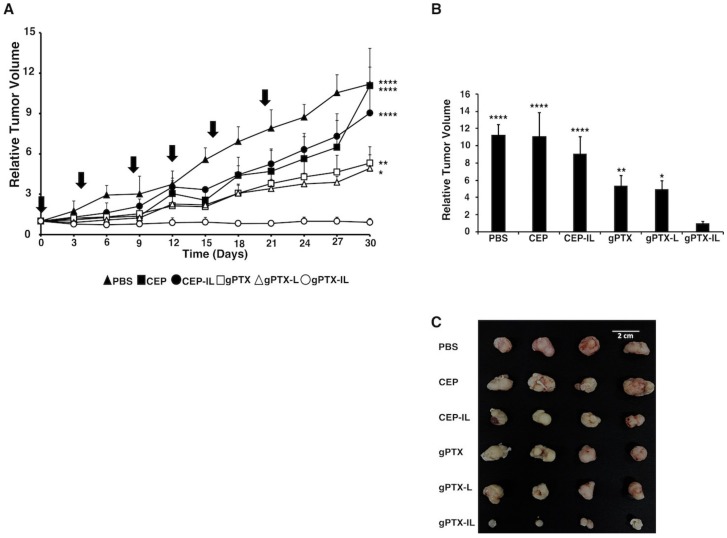
gPTX-IL suppressed tumor growth in the most effective manner in vivo. gPTX-IL (open circle), gPTX-L (open triangle), naked gPTX (open square), CEP-IL (immunoliposome of CEP solvent) (closed circle), Cremophor EL®, ethanol, and phosphate buffered saline (PBS) (CEP, closed square), or PBS (cross) was intravenously injected at day 0, 4, 8, 12, 16, and 20 (indicated by vertical arrows). (**A**) The effect of different formulations of gPTX on the volume of tumors. (**B**) Relative tumor volume at day 30. gPTX-IL was the most effective formulation to suppress the growth of tumor. The statistical significance in mean values of more than two groups was determined using one-way analysis of variance (ANOVA) and Dunnet multiple comparison test using relative tumor volume of gPTX-IL treatment as a control, (*) *p* < 0.05; (**) *p* < 0.01; (****) *p* < 0.001. (**C**) The tumors from the experiment (**A**) representing each group were displayed exhibiting the effect of each formulation of gPTX. Data are expressed as the mean with ± SD where *n* = 4.

The suppression of tumor growth by gPTX-IL was evaluated in BALB/c nude mice bearing tumors of transplanted SK-OV-3 cells (Figure 7).

**Figure 8:**
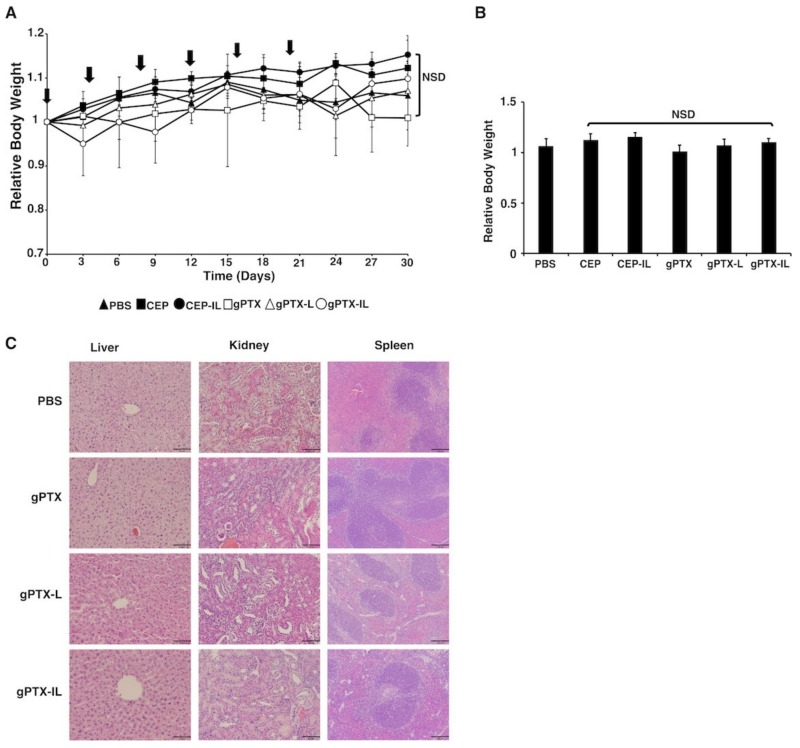
gPTX-IL treatment showed no significance side effects. gPTX- IL (open circle with line), gPTX-L (open triangle), naked gPTX (open square), CEP-IL (closed circle), CEP (closed square), or PBS (cross) was intravenously injected at day 0, 4, 8, 12, 16, and 20 (indicated by vertical arrows). (**A**) Change of body weight of mice bearing tumors. (**B**) Relative body weight at day 30. The statistical significance in mean values of more than two groups was determined using one-way analysis of variance (ANOVA) and Dunnet multiple comparison test using relative body weight of PBS treatment as a control, NSD, no significant difference. (**C**) H & E staining of some vital organs of the drug treated animal groups, each scale bar of liver and kidney section shows 64 µm and spleen section shows 129 µm.

Naked gPTX, gPTX-L, and gPTX-IL were injected intravenously (i.v.) 6 times at the dose of 50 mg/kg of gPTX with 4-day intervals (Figure 7 and Figure 8). We administered PBS, CEP, and CEP-IL (immunoliposome of CEP solvent) as control. PBS used to suspend liposome was injected in equivalence to the maximum volume of injected liposome as the background. CEP as the solvent of naked gPTX was injected in equivalence to the gPTX volume. CEP-IL as the representation of immunoliposome conjugated to anti-hCD44 MAb, which encapsulates CEP alone, was injected based on the amount of lipid equivalent to that contained in gPTX-IL at dose 50 mg/kg. The tumor growth was observed for 30 days and the relative tumor volume was measured at every 3-days, then being normalized to the initial tumor volume at day 0. In our previous report, gPTX-IL conjugated to anti-HER2 MAb, was administered at 150 mg/kg as single dose injection in nude mice bearing HT-29 tumors and targeted with gPTX, it injected for 3 times in the 10 days interval [11]. This was an experiment to investigate the maximum tolerated dose to demonstrate effective high dose administration was possible. In contrast, we evaluated the effect of repeated i.v. injection of gPTX equivalent to 50 mg/kg in this study. All tumors from mice treated with five different formulations were excised at day 30 and were compared by tumor size and volume (Figure 7A–C). Loss of body weight was not observed for any of the formulations (Figure 8A,B). Moreover, pathological observations of liver, kidney, and spleen showed that gPTX-IL did not cause significant damage to the tissues during the experiment while naked drug and non-targeting liposome appeared to damage the tissues (Figure 8C). In gPTX treatment, liver showed a cytoplasmic vacuolation. Simultaneously, the kidney exhibited atrophied glomeruli and necrotic areas. In addition, spleen had enlargement of lymphoid follicles of white pulp. In gPTX-L treatment, elongated trabeculus were found in the spleen. Meanwhile, no damage was found in any of those tissues in gPTX-IL treatment. Of note, the tumor growth appeared completely suppressed solely by the administration of gPTX-IL. As a result, gPTX-IL showed excellent anti-tumor activity with fewer side effects.

## 3. Discussion

In this study, we designed a drug-delivery system targeting ovarian cancer overexpressing CD44. CD44 is known as common CSC marker and considered critically related with the migration and adhesion of CSCs and during the formation of tumor tissue [20]. CSCs are thought to be resistant to chemotherapy and responsible for metastasis and recurrence. CD44 positive epithelial ovarian cancer stem cells were also found to be correlated with drug resistance and recurrence [22,27]. Therefore, we consider that the drug delivery targeting CD44 in ovarian cancer could lead to successful results in suppressing specifically CSC population in the tumor site. The screening of three ovarian cancer derived cell lines showed SK-OV-3 cells overexpressing CD44 while CD44 expression in OVCAR-3 and OVK18 cells was less or deficient (Figure 1). Consequently, SK-OV-3 cells were selected as the target of the drug delivery. Double staining of CD44 and CD24, which are both correlated with CSC markers, exhibited two different populations of CD44^+^/CD24^−^ and CD44^+^/CD24^+^ within SK-OV-3 cells, and only CD44^−^/CD24^+^ in OVCAR-3 cells and CD44^−^/CD24^−^ in OVK18 cells (Figure 2). The phenotype of CD44^+^/CD24^−^ in ovarian cancer cells has been reported to exhibit CSC-like properties of enhanced differentiation, invasion, and resistance to chemotherapy [28,29], while CD44^+^/CD24^+^/Epcam^+^ exhibited stem cell characteristics in other reports [30]. Therefore, SK-OV-3 cells display a phenotype which should be a suitable candidate for the purpose to design drug delivery targeting CSC-like population in ovarian cancers.

In the present study, we successfully improve the encapsulation efficiency of both gPTX-L and gPTX-IL up to approximately 70%–90% (Table 1) by replacing the process of down-sizing liposome from sonication to extrusion. The liposomes diameter of approximately 100 nm of gPTX-L and gPTX-IL could be practical for the drug delivery i.v. since particle size between 50 and 200 nm is considered sufficient for the accumulation of the drug in tumor via enhanced permeability and retention effect (EPR) [31].

The design of targeting CD44 is already employed to enhance drug efficacy and reduce systemic toxicity in ovarian cancer [32,33]. In this study, we employed anti-hCD44 MAb to design the liposome to target ovarian cancer. FAM-IL could successfully allow FAM uptake in the CD44 positive ovarian cancer in one hour exposure of FAM-IL (Figure 4). This result suggests that CD44-targeted liposomes involve receptor-mediated endocytosis with additional membrane fusion of lipid bilayers, resulting in a higher cellular uptake, as previously described that CD44 upregulation in the cancer cells assisted endocytosis via independent clathrin-coated vesicles, the caveolae or macropinocytosis pathway [34,35,36]. As a result, gPTX-IL found to exhibit the most efficient target-oriented cytotoxicity in SK-OV-3 cells when compared to gPTX-L and naked gPTX in 24-h treatment (Figure 6). We further evaluated the antitumor effects of gPTX-L and gPTX-IL in vivo with repeated administration at a total dose of 300 mg/kg gPTX. gPTX-IL exhibited the most effective antitumor activity without side effects, which was evaluated by a loss of body weight and H&E staining of liver, kidney, and spleen (Figure 8). These results are consistent with those previously reported: a liposome conjugated to anti-HER2 antibody efficiently accumulated and internalized into the HER2 positive cells in tumor tissue while non-targeting liposomes were localized in the stroma [11,37]. In this context, gPTX-IL is conceivable superior to gPTX-L in the inhibition of tumor growth due to the retention time in tumor site.

We included CEP-IL as additional control in the in vivo experiment to see the role of CD44 antibody intrinsically, as the stated antibody bound to an empty liposome is able to improve the therapeutic efficacy and inhibit tumor growth even without cancer drug [38]. CEP-IL did not show significant inhibition on tumor growth, although antigen-dependent cellular cytotoxicity should be expected by the phagocytic activation of macrophages targeting the IgG molecule bound to antigens.

In summary, gPTX-IL was successfully demonstrated reduction of tumor volume and the therapeutic efficacy against CD44-overexpressing ovarian cancer cells in vivo. Therefore, gPTX-IL should have a potential as an advantageous strategy of drug delivery targeting cell surface molecules specifically to ovarian cancer cells.

## 4. Materials and Methods

### 4.1. Materials

Dipalmitoylphosphatidylcholine (DPPC), 1,2-distearoyl-sn-glycerol-3-phosphoethanolamine-N-[methoxy(polyethylene glycol)-2000] (mPEG–DSPE), and 1,2-distearoyl-sn-glycerol-3-phosphoethanolamine-N-[maleimide (polyethylene glycol)-2000] (Mal–PEG–DSPE) were obtained from NOF Co. (Tokyo, Japan). Cholesterol (Chol) was purchased from Kanto Chemical Co., Inc. (Tokyo, Japan). Thiazolyl blue tetrazolium bromide (MTT), RPMI 1640 medium and Dulbecco’s modified Eagle’s medium (DMEM) were obtained from Sigma-Aldrich (St Louis, MO, USA). gPTX was synthesized as previously described [12].

### 4.2. Cell Culture and Experimental Animal

The human ovarian cancer cell lines SK-OV-3 cells (HTB-77, ATCC, Manassas, VA, USA ) and OVK18 cells (TKG 0323, Cell Bank, Tohoku University, Sendai, Japan) were cultured in DMEM supplemented with 10% fetal bovine serum (FBS) (Thermo Fisher Scientific, Waltham, MA, USA), containing 100 U/mL penicillin (Nacalai tesque, Kyoto, Japan), and 100 μg/mL streptomycin and OVCAR-3 (HTB-161, ATCC, Manassas, VA, USA) were cultured in RPMI 1640 medium supplemented with 10% FBS containing 100 U/mL penicillin, and 100 μg/mL streptomycin.

Four-week-old female BALB/c nude mice from Charles River (Kanagawa, Japan) were bred at 23 °C and fed with sterilized food and water during the experiments. All animal experimental protocols were reviewed and approved by the ethics committee (Animal Care and Use Committee) of Okayama University under the project identification code IDs OKU-2016078 (Date of approval: 1 April 2016).

### 4.3. Preparation of Anti-hCD44 MAb

To produce anti-hCD44 MAb, hybridoma Hermes-3 cells (HB-9480, ATCC, Manassas, VA, USA) cells were cultured using a bioreactor, miniPERM (SARSTEDT, Nümbrecht, Germany). Twenty million of the cells were suspended in 50 mL of PFHM-II (Gibco, Grand Island, NY, USA) medium and were transferred into production module. The production module was connected to nutrient module containing 350 mL of PFHM-II. The bioreactor was rotated for 10 days at 37 °C in 5% CO2. The medium in the production module was then collected and centrifuged at 150× *g* for 5 min at 4 °C to remove the cells. The supernatant was re-centrifuged at 10,000× *g* for 5 min at 4 °C. The supernatant was then passed through a 0.20 µm filter (Sartorius Stedim Biotech GmBH, Geottingen, Germany) to completely remove cell debris. Anti-hCD44 MAb was then purified as follows: the supernatant was passed through a 0.5 mL of Protein A Sepharose (GE Healthcare, Uppsala, Sweden) equilibrated with PBS. After washing the column with PBS, anti-hCD44 MAb was eluted using 0.1 M sodium-acetic buffer at pH 2.6. Five hundred µL of each fraction was readily neutralized with 10 µL of 2 M sodium phosphate buffer, pH 8.0. The fraction containing anti-hCD44 MAb was detected by Western blotting using polyclonal anti mouse IgG HRP (DAKO, Glostrup, Denmark) and the protein concentration was determined using a BCA assay kit (Pierce Biotechnology, Rockford, IL, USA). Characterization of anti-hCD44 MAb could be find in Figures S1 and S2. 

### 4.4. Expression of CD44 in Ovarian Cancer Cell Line

#### 4.4.1. Western Blotting

Proteins following the SDS-PAGE were transferred to polyvinylidene difluoride (PVDF) membranes (Merck Millipore, Burlington, MA, USA). To detect CD44 epitope, the blot was probed using anti-hCD44 MAb (2 mg/mL, 1:2000) followed by polyclonal anti-mouse IgG HRP (1:4000) (DAKO, Glostrup, Denmark). Quantitative assessment of relative intensity of the blots were analyzed using ImageJ (https://imagej.nih.gov/ij/). The actin immunoreact to anti-beta actin Rabbit MAb (1:1000, 4970S, Cell Signalling Technology, Inc., Beverly, MA, USA) was used as a normalization control.

#### 4.4.2. RNA Isolation and Reverse Transcriptional Quantitative Polymerase Chain Reaction (RT-qPCR)

RNAeasy Mini kit (QIAGEN, Hilden, Germany) was used to isolate total RNA from cells and the extracted RNA was treated with DNase I (Promega, Fitchburg, WI, USA). One µg of RNA was reverse transcribed using GoScript™ Reverse Transcription System (Promega, Fitchburg, WI, USA). qPCR assays were done by LightCycler 480 II (Roche Diagnostics GmbH, Mannheim, Germany) using LightCycler 480 SYBR green I Master Mix (Roche Diagnostics GmbH, Mannheim, Germany) according to the manufacturer’s instructions. Gene expression level was normalized with glyceraldehyde-3-phosphate dehydrogenase GAPDH mRNA. The primers used for the reverse transcriptional quantitative polymerase chain reaction (RT-qPCR) analysis are listed in Table S1.

#### 4.4.3. Flow Cytometry Analysis

SK-OV-3 cells and OVK18 cells were harvested at logarithmic growth phase, followed by being re-suspended in 100 µL PBS, stained with APC (Allophycocyanin)-labelled mouse anti-human CD44 MAb (BD Science Pharmingen, San Diego, CA, USA) and FITC (fluorescein isothiocyanate )-labelled mouse anti-human CD24 MAb (BD Science Pharmingen, San Diego, CA, USA), and analyzed by BD AccuriTM C6 plus flow cytometer (Becton & Dickinson, Franklin Lakes, NJ, USA). Data of each experiment was analyzed using FlowJo software (FlowJo LLC, Ashland, OR, USA).

### 4.5. Preparation of Liposome Encapsulating gPTX

#### 4.5.1. Preparation of gPTX-L

This preparation was conducted with modified of previous method [11]. Liposomes composed of DPPC and cholesterol (by ratio 3:1) with 5 mol% mPEG–DSPE were prepared by the thin-film hydration method. In brief, DPPC and Chol with 5 mol% mPEG–DSPE were dissolved in an organic solvent of chloroform/methanol (9:1 *v*/*v*) in an egg flask. The flask was connected to a rotary evaporator (Eyela, Shanghai, China), which was maintained at 50 °C under aspirator vacuum. The resulting lipid film was left overnight under vacuum to remove remaining organic solvent. The fully dehydrated lipid film was suspended in CEP (Chremophor, ethanol, PBS in 12:12:76 volume ratio) by vortexing at 60 °C, resulting in the formation of multilamellar vesicles (MLVs).

MLVs were frozen and thawed for five cycles. A single freeze-thaw cycle consisted of freezing at −196 °C liquid nitrogen for 1 min and thawing at 55 °C water bath for 1 min. The liposomes were then extruded 10 times through a single stack of one 100 nm Whatman polycarbonate membrane (GE Healthcare, Carlsbad, CA, USA) using the Mini Extruder (Avanti Polar Lipids, Inc., Alabaster, AL, USA) to form small lamellar vesicles (SLVs). The extruder was kept warm at 55 °C on a hot plate prior to extrusion. The outer solvent of the liposomes was replaced CEP with PBS by ultrafiltration with a 100K-membrane filter (Merck Millipore Ltd., Billerica, MA, USA) at 5000× *g* for 20 min five times. Then, gPTX (1 mg/mL) in 40% EG was added into the solution of liposome encapsulating CEP (CEP-L) at 60 °C. gPTX-L was then concentrated to the volume before added drug by ultrafiltration. This encapsulation process was conducted three times. Finally, residual gPTX was removed by washing the liposomes with PBS followed by ultrafiltration at 5000× *g* for 20 min five times.

#### 4.5.2. Preparation of gPTX-IL

CEP-L composed of DPPC and Chol (by ratio 3:1) with 4 mol% mPEG–DSPE was incubated with 0.5 mol% Mal–PEG–DSPE at 50 °C for 10 min to introduce maleimide functional groups into liposome to conjugate antibodies. Then, gPTX was encapsulated using the solubility gradient method described above. To immobilize antibody on the surface of the liposomes, SH groups were introduced into anti-hCD44 MAb by treatment with 2-iminothiolane (Sigma–Aldrich, St. Louis, MO, USA) at a molar ratio of 1:50 in 25 mM HEPES, pH 8.0 containing 140 mM NaCl. The mixture was subsequently incubated for 1 h at room temperature in the dark. After removing unreacted 2-iminothiolane gel filtration with a G25 PD-10 column (GE Healthcare, Uppsala, Sweden), the modified anti-hCD44 MAb was incubated with liposomes containing Mal–PEG–DSPE overnight at 4 °C. To block free maleimide groups, liposomes were then incubated with L-Cystein (0.5 mM final concentration) for 15 min at 25 °C. Residual L-Cystein was removed by ultrafiltration with a 100K-membrane filter 5000× *g* for 20 min for five times and followed by removing free anti-hCD44 MAb by ultrafiltration with a 300K membrane filter (Sartorius Stedim Biotech GmbH, Gottingen, Germany) at 6000× *g* for 20 min at 4 °C.

### 4.6. Evaluation of Cellular Uptake

#### 4.6.1. Preparation of Fluorescent Liposome

Lipid composition and hydration step as same as gPTX-L preparation for fluorescent liposome. gPTX replaced by solution of 5 mmol 6-Carboxyfluorescein (FAM) (Molecular Probes Inc, Eugene, OR, USA) in PBS. The fully dehydrated lipid film was suspended by the FAM solution in PBS to produce FAM-liposome (FAM-L) by the direct encapsulation. To prepare FAM Immunoliposome (FAM-IL), Anti-CD44 MAb was conjugated by the same procedure used for conjugation in gPTX-IL.

#### 4.6.2. Confocal Microscopic Observation

SK-OV-3 cells and OVK18 cells were seeded on gelatin-coated 18 mm coverslip (Iwaki, Japan) in 12-well plates. The cells were incubated with 1 μM FAM-L and FAM-IL in serum-free medium for 2 h at 37 °C in an atmosphere of 5% CO_2_. The cells were washed three times with cold PBS and fixed with 4% paraformaldehyde in PBS. The coverslips were placed on the slide that already mounted with mounting-solution reagent containing DAPI (Vector Lab, Burlingame, CA, USA) then visualized under a confocal microscope (Fluoview FV-1000, Olympus, Tokyo, Japan).

#### 4.6.3. Flow Cytometry Observation

SK-OV-3 cells and OVK18 cells were seeded 1 × 10^5^ cells/well at the 12-well plates. After incubation at 37 °C in 5% CO_2_ for 24 h, 1 μM FAM-L and FAM-IL were applied for 1 h and 3 h in serum-free medium. Cells were trypsinized, washed by PBS three times, followed by being re-suspended in 300µl PBS and analyzed by BD Accuri C6 plus flow cytometer (Becton and Dickinson, Franklin Lakes, NJ, USA). Data of each experiment was analyzed using FlowJo^®^ software (FlowJo, LLC, Ashland, OR, USA).

### 4.7. Characterization of Liposome

#### 4.7.1. Size Distribution of Particle and Zeta Potential

The size and zeta potential of liposomes were determined by dynamic and electrophoretic light scattering using an ELS-8000 (Photal Otsuka Electronics, Osaka, Japan).

#### 4.7.2. Evaluation of Encapsulation Efficiency (EE) and Loading Efficiency (LE)

Encapsulation Efficiency (EE) was calculated as the ratio of the amount of gPTX encapsulated into liposomes to the initial amount of the drug. Loading Efficiency (LE) was calculated as the molar ratio of the drug encapsulated into liposomes to the total lipid and chol. The amount of encapsulated drug was evaluated by C18 reverse-phase HPLC (Hitachi Elite LaChrom L-2400, Tokyo, Japan) under an isocratic condition of 60% (*v*/*v*) methanol at a flow rate of 1 mL/min. Ten µL of each sample were injected and the drug was detected at 227 nm.

#### 4.7.3. Transmission Electron Microscopy (TEM)

A 400-mesh copper grid coated with formvar/carbon films was hydrophilically treated. Liposome suspension (5 to 10 μL) was placed on Parafilm, and the grid was floated on that suspension and left for 15 min. The sample was negatively stained with 2% uranyl acetate solution for 2 min. liposome on the grid were visualized with 20,000 times magnification with an H-7650 transmission electron microscope (Hitachi, Tokyo, Japan) at Central Research Laboratory, Okayama University Medical School.

### 4.8. Cytotoxicity Assay

#### 4.8.1. Drug Sensitivity Evaluation

Prior to the evaluation, we tried to optimize the conditions to observe the drug sensitivity. For SK-OV-3 cells, we compared two conditions after exposure of drugs for 72 h. One is to add MTT directly after the exposure and the other is to add MTT following incubation for 48 h after drug exposure (Figure S3). Evaluation of IC_50_ was not possible when MTT was directly added after 72 h of drug exposure. Extended incubation for 48 h without drug just after the treatment allowed us to evaluate the IC_50_. For OVK18 cells the evaluation was possible to evaluate the IC_50_ when MTT was added just after the treatment with drug for 72 h. The extended incubation of further 48 h was not adequate for the evaluation of cytotoxic effect on OVK18 cells because the survived cells reached overgrowth, which affected the cell growth. Drug sensitivity was checked by MTT assay after 72 h (OVK18) and 120 h (SK-OV-3). Cells were seeded in a 96-well plate at 5000 cells/well. After incubation at 37 °C in 5% CO_2_ for 24 h, different concentrations PTX and gPTX were added to each well. For OVK18, after incubation for 72 h, 4.25 mg/mL MTT solution was added at a final concentration of 0.5 mg/mL in each well and the plate was incubated for 4 h. The initially seeded cell number (5000 cells/well) and the drug exposure time (72 h) were fixed in both SK-OV-3 cells and OVK18 cells; therefore, the extended of incubation time should not induce any biases. For SK-OV-3 cells, after incubation for 72 h, the medium with drug was replaced with fresh medium without drug, and incubation were continued until 48 h, 4.25 mg/mL MTT solution was added at a final concentration of 0.5 mg/mL in each well and the plate was incubated for 4 h. Formed formazan crystals were dissolved with 10% (*w*/*v*) SDS in 0.02 N HCl and incubated overnight. Finally, the absorbance of each well was measured at 570 nm using an MTP-800 Lab microplate reader (Corona Electric, Ibaraki, Japan). The experiment was performed in triplicate. IC_50_s were estimated from the survival curve.

#### 4.8.2. Evaluation of Cytotoxic Effects of Liposome Formulation by 24 h and 72 h Treatment

In vitro cytotoxicity was evaluated by the MTT assay after 72 h (OVK18) and 120 h (SK-OV-3) of cells were seeded in a 96-well plate at 5000 cells/well. After incubation at 37 °C in 5% CO_2_ for 24 h, different concentrations of gPTX were added to each well. After incubation of drug for 24 h and 72 h, MTT assay was performed as described above.

### 4.9. Evaluation of Antitumor Effects of Drugs In Vivo

The xenograft of SK-OV-3 cells in mice was prepared by a subcutaneous injection of 7.5 × 10^6^ cells/mouse. Tumor volume was measured by a Vernier caliper and calculated as [length × (width)^2^]/2. The anti-tumor effect of each formulation was evaluated when the tumor volume reached 50–200 mm^3^. Mice were randomly assigned to five groups *(n* = 4); group 1 for PBS, group 2 for CEP, group 3 for CEP-IL, group 4 for naked gPTX, group 5 for gPTX-L and group gPTX-IL; 50 mg of gPTX-equivalent per kg body weight was injected six times via tail vein at the intervals of 4 days. Tumor volumes and body weights were measured at 3- or 4-day intervals. Paraffin-embedded liver, kidney, and spleen sections (5-μm thick) were stained with Hematoxylin (Sigma Aldrich, St. Louis, MO, USA) and 0.5% Eosin Y (Wako, Osaka, Japan) (HE) for histological analysis then visualized under a FSX100 Inverted Microscope (Olympus, Tokyo, Japan).

### 4.10. Statistical Analysis

All the experiments were repeated at least three times. Data were depicted as means ± standard deviation. The statistical significance in mean values between two groups was determined by 2-tailed student’s *t*-test. The statistical significance between the mean values of more than two groups was determined using one-way analysis of variance (ANOVA) and Dunnett’s multiple comparisons test. *p* < 0.05 was considered statistically significant.

## 5. Conclusions

The cytotoxicity of gPTX, a derivative of PTX with a glucose moiety, exhibited the cytotoxicity equivalent of one fifth of the cytotoxicity by PTX against ovarian cancer-derived cells. This cytotoxicity was considered due to the hydrophilicity from the glucose moiety. However, the IC_50_ of gPTX toward the cells was found to be still low enough to target ovarian cancer. Down-sizing of liposome by extruder and supplementing cysteine-masking unreacted radicals improved the procedure of immunoliposome preparation to achieve practical encapsulation efficiency for gPTX-L and gPTX-IL. As a result, the preparation of sufficient quantities of both gPTX-L and gPTX-IL became practical.

gPTX-IL should quickly recognize the CD44 positive cancer cells and retain on the cell surface due to the antibody, implying that the potential therapeutic effect should be high. As expected, gPTX-IL exhibited distinguished inhibition of tumor growth with less apparent side effects in vivo. Targeting CD44 in the ovarian cancer should be attributed to targeting the CSC population, since overexpression of CD44 in SK-OV-3 could correlate with CSC–like character. Taking these into consideration, the immunoliposomes encapsulating a practical amount of gPTX should be a promising formulation for anticancer drugs as a positive targeting drug delivery system in ovarian cancer.

## Figures and Tables

**Table 1:** Characters of the liposomes encapsulating gPTX.

Formulation	Diameter (nm)	Polydispersity Index	Zeta Potential (−mV)	Encapsulation Efficiency (%)	Loading Efficiency (%)
gPTX-L	115 ± 29	0.20 ± 0.02	6.9 ± 1.5	86.8 ± 10.1	9.4 ± 1.1
gPTX-IL	99.8 ± 12	0.26 ± 0.01	7.8 ± 1.2	80.9 ± 10.6	8.9 ± 1.2
*t*-test	NSD	*	NSD	NSD	NSD

Each experiment was performed in triplicate and the values are given as mean ± SD. The statistical significance in mean values between gPTX-L and gPTX-IL was performed by two-tailed students *t*-test. * *p* < 0.05; NSD, no significant difference.

## Supplementary Materials

Supplementary Materials can be found at https://www.mdpi.com/1422-0067/20/5/1042/s1.

## Abbreviations

PTX	Paclitaxel
gPTX	Glycosylated paclitaxel
anti-hCD44 MAb	Anti-human CD44 monoclonal antibody
gPTX-L	Glycosylated paclitaxel-liposome
gPTX-IL	Glycosylated paclitaxel-immunoliposome (anti-hCD44 MAb)
FAM	6-Carboxyfluorescein
FAM-L	6-Carboxyfluorescein-Liposome
FAM-IL	6-Carboxyfluorescein-Immunoliposome (anti-hCD44 MAb)
CEP	Cremophor, ethanol, PBS (12:12:76 volume ratio)
CEP-IL	Cremophor, ethanol, PBS-Immunoliposome (anti-hCD44 MAb)
PBS	Phosphate buffered saline
