# Supplementary material for: Targeting Ovarian Cancer Cells Overexpressing CD44 with Immunoliposomes Encapsulating Glycosylated Paclitaxel
Source: Int J Mol Sci. 2019 Feb 27;20(5):1042. doi: 10.3390/ijms20051042 (PMC6429518; doi:10.3390/ijms20051042)
Supplement: Supplementary file 1 [file ijms-20-01042-s001.pdf]

International Journal of *Molecular Sciences*

MDPI

Supplementary Materials: Targeting Ovarian Cancer Cells Overexpressing CD44 with Immunoliposomes Encapsulating Glycosylated Paclitaxel

Apriliana Cahya Khayrani[1], Hafizah Mahmud[1], Aung Ko Ko Oo[1], Maram H. Zahra[2], Miharu Oze[1], Juan Du[1], Md Jahangir Alam[1], Said M Afify[1], Tsukasa Shigehiro[1,3], Anna Sanchez Calle[4], Akimasa Seno[2], Hiroki Hamada[5], Yuhki Seno[6,7], Tadakatsu Mandai[7], Masaharu Seno[1,2*]

Table S1. List of primers used in the experiments.

No	Names	Forward primer Sequence (5'->3')	Reverse primer Sequence (5'->3')
1	CD44s	TGGGTTCATAGAAGGGCACG	AGGTGGAGCTGAAGCATTGAA
2	GAPDH	CAACGACCACTTTGTCAAGCTC	GGTCTACATGGCAACTGTGAGG

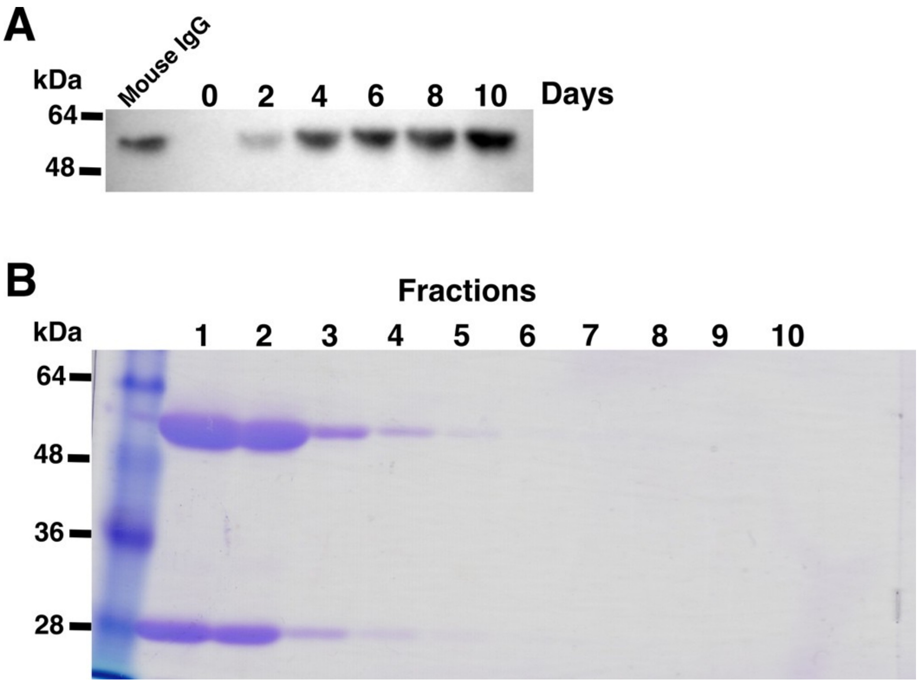

Figure S1. Anti-hCD44 MAb preparation. (A) Western blot result of conditioned medium of HB-9480 cells culture for 0 to 10 days detected by goat anti-mouse IgG-HRP (Santa Cruz Biotechnology Inc., CA, USA), 100 ng of Mouse IgG as positive control. (B) CBB staining of SDS PAGE of anti-hCD44 MAb eluted from protein A column.

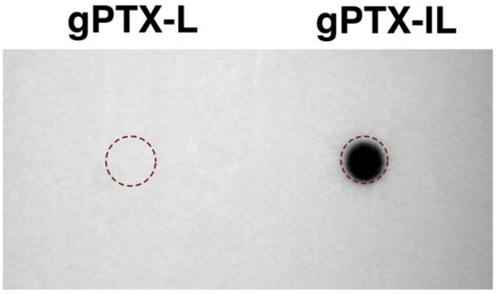

Figure S2. Dot blotting analysis of liposomes conjugated to ligands. Liposomes containing approximately 6 µg of gPTX in 2 µL were blotted onto PVDF membrane and probed with peroxidase-labelled Protein A (KPL, USA) (red dashed circle indicate the positions of dots). The immunoreactivity indicates that anti-hCD44 MAb was conjugated to liposome.

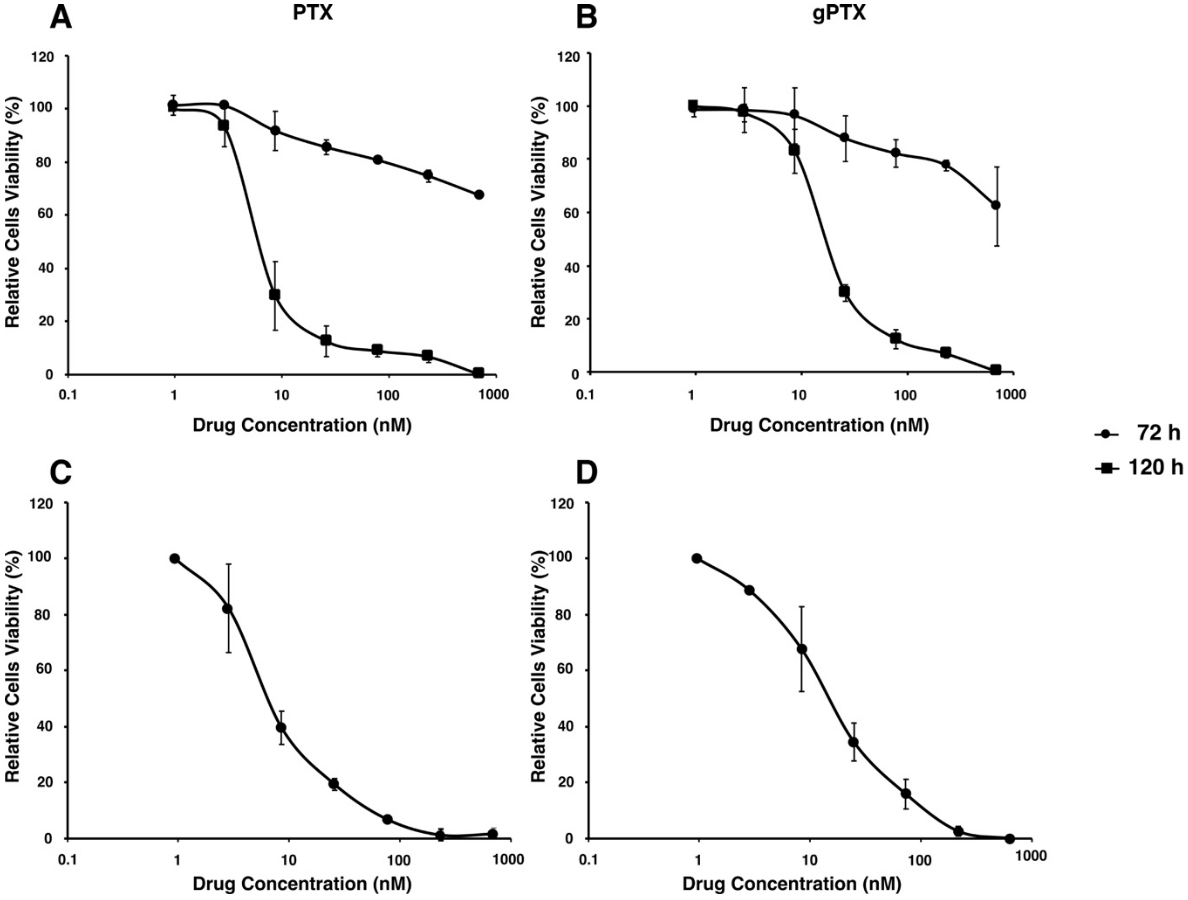

Figure S3. Drug sensitivity evaluation of SK-OV-3 cells (A, B) and OVK18 cells (C,D) after 72 hours drug exposure (closed circle is MTT reagent directly added after drug treatment, closed square is MTT added after 48 hours drug replaced by fresh medium). (A, C) Cells were treated with PTX. (B, D) Cells were treated with gPTX.

© 2019 by the authors. Submitted for possible open access publication under the terms and conditions of the Creative Commons Attribution (CC BY) license (http://creativecommons.org/licenses/by/4.0/).
